# Philadelphia chromosome-positive acute lymphoblastic leukemia with extramedullary and meningeal relapse after allogeneic hematopoietic stem cell transplantation that was successfully treated with dasatinib

**DOI:** 10.1186/2193-1801-3-177

**Published:** 2014-04-05

**Authors:** Toshinori Kondo, Taizo Tasaka, Kana Matsumoto, Rui Matsumoto, Lisa Koresawa, Fuminori Sano, Hirotoshi Tokunaga, Yoshiko Matsuhashi, Hidekazu Nakanishi, Kunihiko Morita, Hideho Wada, Takashi Sugihara

**Affiliations:** Division of Hematology, Kawasaki Medical School, 577, Matsushima, Kurashiki, Okayama, 701-0192 Japan; Department of Clinical Pharmaceutics, Faculty of Pharmaceutical Sciences, Doshisha Women’s College of Liberal Arts, Kodo, Kyotanabe, Kyoto, 610-0395 Japan

**Keywords:** Philadelphia chromosome-positive acute lymphoblastic leukemia, Meningeal leukemia, Extramedullary relapse, Hematopoietic stem cell transplantation, Dasatinib

## Abstract

Central nervous system (CNS) relapse is a critical issue while treating Philadelphia chromosome-positive acute lymphoblastic leukemia (Ph-positive ALL). A 58-year-old woman with Ph-positive ALL who relapsed after bone marrow transplantation for meningeal leukemia was treated with high-dose methotrexate, which resulted in remission. She underwent allogeneic cord blood transplantation followed by reduced intensity conditioning chemotherapy with imatinib; however, she experienced CNS relapse and developed an extramedullary mass on the right side of the temporal region. We treated 40 mg of dasatinib once daily, which had to be temporarily discontinued because she developed grade 2 pleural effusion and grade 2 hematemesis. After reinitiation of dasatinib, the extramedullary mass disappeared and meningeal leukemia ameliorated almost immediately. With 40 mg dasatinib administered once daily, its trough level and cerebrospinal fluid (CSF) concentration were 32 ng/mL and below the sensitivity threshold of 1 ng/mL, respectively. Treatment was continued, and the patient remained in complete remission until she died of pneumonia 7 years after the initial diagnosis of ALL. Dasatinib can be an effective treatment for Ph-positive ALL with CNS relapse. Although the concentration in the CSF seems low, it may be sufficient to exert anti-leukemic effects in the human CNS.

## Background

Philadelphia chromosome-positive acute lymphoblastic leukemia (Ph-positive ALL) is a serious hematological malignancy that usually requires combination chemotherapy to achieve complete remission. Patients with Ph-positive ALL often develop resistance to chemotherapy, which leads to relapse and death. Even with intensive consolidation and maintenance therapies, many patients who achieve remission relapse after a short period of time. Previous studies on Ph-positive ALL patients reported a median disease-free survival (DFS) and 3-year DFS rate of 8.7 months and 24%, respectively, while the 5-year overall survival (OS) rate remained low at 24%–34% (Yanada et al. 2009; Iida et al. 2004; Thomas et al. 2004). Adult ALL patients rarely present with meningeal leukemia at initial diagnosis (only approximately 5% of ALL patients), and central nervous system (CNS) relapse occurs in 5%–10% patients who receive routine CNS-directed prophylactic therapy (Lazarus et al. 2006; Pfeifer et al. 2003). The outcome of patients with meningeal leukemia is worse than that of patients without, despite CNS-directed therapy (Lazarus et al. 2006). In recent years, the oral tyrosine kinase inhibitors (TKIs) imatinib and dasatinib have been prescribed for the treatment of relapsed Ph-positive ALL after hematopoietic stem cell transplantation (HSCT) (Ishida et al. 2010; Czyz et al. 2010; Millot et al. 2009; Takami et al. 2006). TKI combined with conventional chemotherapy and incorporated into the transplantation strategy has improved the long-term survival of patients with Ph-positive ALL and has allowed the de-escalation of chemotherapy. This combination treatment strategy has prolonged DFS and OS (Thyagu et al. 2012; Tanguy-Schmidt et al. 2013) beyond the current rates. Dasatinib is an active agent in heavily pretreated Ph-positive ALL patients, including those who have undergone prior HSCT or those who have been treated previously with imatinib-containing therapy (Takami et al. 2006; Sakamaki et al. 2009; Ottmann et al. 2007; Tachibana et al. 2011). Dasatinib appears to reach the cerebrospinal fluid (CSF) better than other TKIs according to recent reports that claimed stabilization and regression of CNS disease in a small series of patients (Alimena et al. 2009; Porkka et al. 2008; Abdelhalim et al. 2007). The CSF concentration of dasatinib after oral administration in humans is poorly understood. Here we describe the case of a Ph-positive ALL patient who developed extramedullary relapse with CNS involvement after imatinib-based treatment and subsequent HSCT. The patient exhibited a complete response to dasatinib-based therapy, which resulted in long-term survival. We also investigated the dasatinib concentrations in this patient’s plasma and CSF.

## Case description

A 58-year-old woman presented with loss of appetite and general fatigue. Blood examination revealed marked leukocytosis (37.6 × 10^9^/L) with 81.5% lymphoblasts. Bone marrow aspiration showed that 95% lymphoblastic cells were positive for B-cell markers, including CD10, CD19, CD34, and human leukocyte antigen (HLA)-DR, while cytogenetic analysis reported a complex karyotype including t(9;22) (q34;q11). Real-time quantitative polymerase chain reaction (RT-qPCR) detected 3.1 × 10^5^ copies/μg of RNA *p210 bcr-abl* transcripts in the marrow specimen. The patient was diagnosed with Ph-positive precursor B-cell ALL in April 2005, when imatinib-combined induction therapy was initiated (Yanada et al. 2006).

She exhibited complete hematological and cytogenetic responses, and *bcr-abl* transcripts were negative according to RT-qPCR. In July 2005, after receiving high-dose methotrexate (MTX) therapy as CNS prophylaxis, she underwent bone marrow transplantation (BMT) using an allogeneic bone marrow graft from an HLA-matched sibling donor after a conditioning regimen with fludarabine (25 mg/m^2^/day for 5 days), busulfan (2mg/kg/day for 2 days), and melphalan (80 mg/m^2^/day for 1 day). Cyclosporine A and short-term MTX were used as prophylaxis against graft-versus-host disease (GVHD). The patient exhibited rapid and sustained engraftment, with a neutrophil count higher than 0.5 × 10^9^/L and a platelet count higher than 50 × 10^9^/L on day +16. However, 3 months after BMT, she relapsed with meningeal leukemia, despite being treated with prophylactic intrathecal chemotherapy before BMT. She was subsequently administered high-dose MTX therapy and 6 cycles of MTX-based intrathecal chemotherapy. This regimen eliminated lymphoblastic cells from her CSF, but 1.6 × 10^5^ copies/μg of RNA *p210 bcr-abl* transcripts were still detected in her marrow blood. In July 2006, she underwent allogeneic cord blood transplantation (CBT) after a conditioning regimen that included fludarabine (30 mg/m^2^/day for 5 days), cytarabine (1.5 g/m^2^/day for 4 days), melphalan (80 mg/m^2^/day for 1 day), and total body irradiation with 4 Gy. Prophylaxis against GVHD was performed with continuous infusion of tacrolimus.

**Figure 1:**
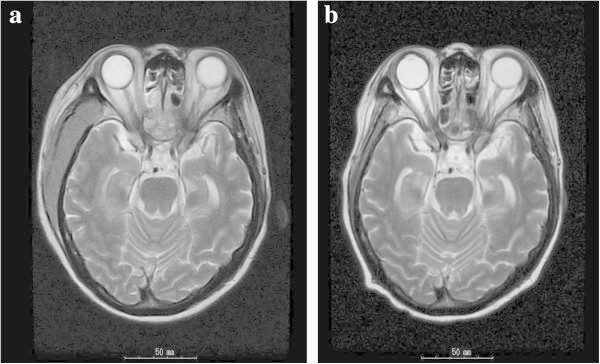
**MRI images of the head. a** The extra-medullary mass of right temporal region before dasatinib treatment. **b** Forty-six days after initial dasatinib treatment.

Neutrophil engraftment was observed on day +18, but acute GVHD was not observed. The patient developed a limited type of chronic GVHD on day +165; nevertheless, she responded well to treatment with prednisolone. However, 7 months after CBT, she relapsed again, developing meningeal leukemia accompanied by headache. Imatinib and intrathecal chemotherapies were initiated again, and whole-brain irradiation (24 Gy in total) was added to her treatment regimen. She achieved remission, and imatinib therapy was continued to prevent CNS relapse. In April 2009, she again complained of headache, and cranial magnetic resonance imaging revealed an extramedullary mass on the right side of the temporal region (Figure 1a).

**Figure 2:**
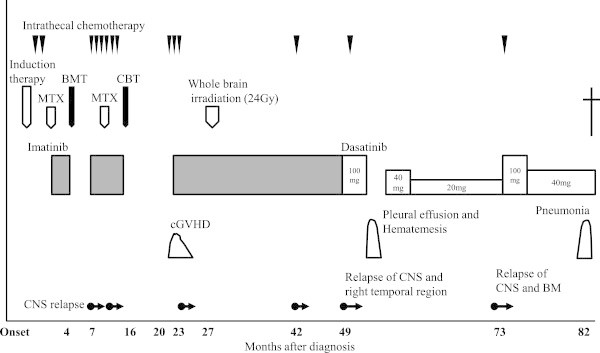
**Clinical course of the patient after diagnosis.** X-axis shows months after diagnosis. MTX; methotrexate, BMT; allogeneic bone marrow transplantation, CBT; allogeneic cord blood transplantation, cGVHD; chronic graft versus host disease.

The clinical course of the patient after relapse is shown in Figure 2. Because of molecular resistance against imatinib, the patient was treated with 100 mg of dasatinib daily and was administered 2 cycles of intrathecal chemotherapy. While on dasatinib therapy, the patient, despite receiving adequate supportive therapy, experienced grade 2 pleural effusion and grade 2 hematemesis according to the National Cancer Institute Common Terminology Criteria (NCI-CTC). Dasatinib was discontinued because of these adverse events and reinitiated at a daily dose of 40 mg once she recovered. The extramedullary mass in her temporal region disappeared (Figure 1b), and meningeal leukemia was ameliorated almost immediately. During the course of treatment, we investigated the dasatinib concentrations in the patient’s plasma and CSF using high-performance liquid chromatography coupled with electrospray mass spectrometry (HPLC-MS) as described previously (De Francia et al. 2009), albeit with some modifications. The trough level and CSF concentration of dasatinib administered at a daily dose of 40 mg were 32 ng/mL and below the sensitivity threshold of 1 ng/mL, respectively. Two months after the initiation of dasatinib treatment, large granular lymphocytosis was observed in the peripheral blood. These large granular lymphocytes were CD3, CD8, CD56, and CD57 positive, and PCR revealed an oligoclonal pattern of T-cell receptor gene rearrangement (data not shown). Until April 2011, the patient remained in complete remission while taking 40 mg of dasatinib once daily; however, meningeal relapse was revealed after close investigation of vertigo. Bone marrow examination showed the presence of 73 copies/μg of RNA *p210 bcr-abl* transcripts that were detected by RT-qPCR. In addition, the lymphoblasts in her CSF harbored an E255K ABL tyrosine kinase domain mutation, which is known to be moderately resistant to both dasatinib and nilotinib. On the basis of these results, we increased the dose of dasatinib to 100 mg once daily and administered one cycle of intrathecal chemotherapy. Three weeks after the initiation of this treatment, the patient achieved complete molecular remission in the bone marrow, and lymphoblasts were undetectable in the CSF. Seven years after the initial diagnosis of Ph-ALL, the patient died of pneumonia. Until the time of her death, no evidence of ALL recurrence was observed.

## Discussion and Evaluation

CNS leukemia in patients with adult ALL is an uncommon finding at initial diagnosis, affecting only approximately 5% of adult ALL patients. The 5-year OS rate of these patients is lower than that of adult ALL patients without CNS leukemia (29% vs 35%, respectively) (Lazarus et al. 2006), and allogeneic HSCT is considered the only curative option for these high-risk patients. However, relapse remains the main cause of treatment failure. The relapse rate of ALL patients with CNS leukemia who achieve complete remission after allogeneic HSCT was reported to be approximately 20%. A third of these patients develop recurrent CNS leukemia if they relapse (Lazarus et al. 2006). Although relapse after HSCT, particularly in the CNS, is a major problem in the management of Ph-positive ALL, effective therapeutic modalities for the prevention of CNS relapse have not been established till date. We observed the long-term survival of a patient with Ph-positive ALL who had been treated with dasatinib for relapsed meningeal leukemia after HSCT. Recently, encouraging reports for the treatment of Ph-positive ALL with TKIs combined with chemotherapy or HSCT were published (Ishida et al. 2010; Czyz et al. 2010; Millot et al. 2009; Takami et al. 2006; Thyagu et al. 2012; Tanguy-Schmidt et al. 2013; Sakamaki et al. 2009; Ottmann et al. 2007; Tachibana et al. 2011; Abdelhalim et al. 2007; Yanada et al. 2006; Nishii et al. 2007; Foa et al. 2011). These reports suggest that dasatinib has strong anti-leukemic activity against Ph-positive ALL, without the prevention of donor hematopoiesis. Therefore, the use of dasatinib as salvage therapy as well as prophylaxis against relapse may be effective for Ph-positive ALL patients who undergo allogeneic HSCT. Dasatinib has been shown to be effective in the treatment of extramedullary leukemia with CNS involvement in Ph-positive leukemia patients (Alimena et al. 2009; Abdelhalim et al. 2007). Porkka et al. reported that dasatinib exerts antitumor effects in a mouse model of intracranial chronic myeloid leukemia and that this drug has substantial benefits for patients with CNS Ph-positive leukemia (Porkka et al. 2008). Dasatinib is thought to penetrate the blood–brain barrier better than imatinib, and this can result in dasatinib having better antileukemic effects against CNS leukemia compared with imatinib. In addition, dasatinib is active at lower concentrations (Porkka et al. 2008), and because the CSF has a lower protein concentration compared with blood, this drug is likely to exist as a free drug in the CNS. However, few reports have examined the concentration of dasatinib in human CSF samples (Porkka et al. 2008). The concentrations of dasatinib in our patient’s plasma and CSF were 32 ng/mL and less than the sensitivity threshold value, respectively, when she was treated with a daily dose of 40 mg. The plasma concentration of 32 ng/mL at trough was higher than that previously reported (Wang et al. 2013), and the discrepancy may be caused by the companion agents administered to our patient, such as azoles, which interfere with the metabolism of dasatinib. Previous studies reported that the dasatinib concentration in the CSF varies between 5% and 28% of the plasma concentration (Porkka et al. 2008). At the oral dasatinib dose of 140 mg once daily or 70 mg twice daily, the CNS concentration was undetectable in 87% patients, even if the samples were collected in the absorptive phase, that is, at 1–4 h after consuming the drug (Porkka et al. 2008). The undetectable dasatinib concentration in the CNS fluid in our patient may indicate a low concentration and an inadequate sampling time at trough. Careful considerations for the timing of sampling of CNS specimens should be made in future studies. Taken together, these results suggest that the concentration of dasatinib in the CSF is usually maintained in a low range, but this low concentration may be enough to exert an anti-leukemic effect in the human CNS. The validity of prophylactic or preemptive therapy with dasatinib after allogeneic HSCT for Ph-positive ALL remains unclear. Till date, 2 studies, each with a small number of cases, have reported encouraging results (Caocci et al. 2012; Teng et al. 2013), suggesting that dasatinib is highly effective in preventing molecular relapse and eradicating Ph-positive ALL after HSCT. At least, prophylactic and preemptive dasatinib therapy should be considered for high-risk patients who do not exhibit molecular responses before or after HSCT and for patients who have extramedullary leukemia with CNS involvement at the time of diagnosis. Prospective and randomized studies aiming to identify the appropriate dasatinib treatment regimen for Ph-positive ALL after allogeneic HSCT are required.

## Conclusion

Dasatinib can be an effective treatment for Ph-positive ALL with CNS relapse.

## Consent

Written informed consent was obtained from the patient for the publication of this report and any accompanying images.
